# Supplementary material for: Complete mitogenome of endemic plum-headed parakeet Psittacula cyanocephala – characterization and phylogenetic analysis
Source: PLoS One. 2021 Apr 9;16(4):e0241098. doi: 10.1371/journal.pone.0241098 (PMC8034733; doi:10.1371/journal.pone.0241098)
Supplement: S1 Table — (DOCX) [file pone.0241098.s001.docx]

| Parameter | Stage1 | Stage 2 | | | Stage 3 | | | Stage 4 | |
| --- | --- | --- | --- | --- | --- | --- | --- | --- | --- |
|  | Incubate | 5 cycles | | | 35 cycles | | | Final | |
|  |  | T_d_ | T_a_ | T_e_ | T_d_ | T_a_ | T_e_ | T_e_ | hold |
| Temperature | 95°C | 95°C | 45°C | 72°C | 95°C | T_a_°C | 72°C | 72°C | 4°C |
| Time (mm:ss) | 05:00 | 00:45 | 00:40 | 01:00 | 00:45 | 00:40 | 01:00 | 10:00 | ∞ |
