# Supplementary material for: Complete mitogenome of endemic plum-headed parakeet Psittacula cyanocephala – characterization and phylogenetic analysis
Source: PLoS One. 2021 Apr 9;16(4):e0241098. doi: 10.1371/journal.pone.0241098 (PMC8034733; doi:10.1371/journal.pone.0241098)
Supplement: S2 Table — (DOCX) [file pone.0241098.s002.docx]

| Sl.No. | Primer name | Sequence | Annealing temperature (°C)  (T_a_) | Predicted amplicon size (bp) |
| --- | --- | --- | --- | --- |
| 1 | PT1F | GGGTTGGTAAATCCTGTGCC | 53 | 1043 |
|  | PT1R | CTCGCTTTGCCTGGTTGTTC |  |  |
| 2 | PT2F | CTACAACACAAAGCATTCAG | 53 | 1027 |
|  | PT2R | TAGCCATTCATACAGGTCTC |  |  |
| 3 | PT3F | CCAAAAACATAGCCTTCAGC | 53 | 767 |
|  | PT3R | TGCCTGGCTCTGCTACACTA |  |  |
| 4 | PT4F | GAAAAGGGTTGCTAGTGTAGC | 53 | 1145 |
|  | PT4R | GATAGGAAATGAGAGGGTTGG |  |  |
| 5 | PT5F | TAGCATACCAATCTCTTACG | 53 | 960 |
|  | PT5R | TCAGGAGGTTATTAGTGTTG |  |  |
| 6 | PT6F | TCACCATAAGCATAACCAAC | 53 | 1057 |
|  | PT6R | CTTATGTTGTTTATGCGTGG |  |  |
| 7 | PT7F | CAGCCCATGCCTTCGTAATA | 53 | 1156 |
|  | PT7R | GGGTAGTCGGAGTATCGTCG |  |  |
| 8 | PT8F | GGCATACCACGACGATACTC | 53 | 952 |
|  | PT8R | TGGACGAGTGGTGATGAATG |  |  |
| 9 | PT9F | CCAATAGAGTCCCCAATCCG | 53 | 860 |
|  | PT9R | GAGGTTGTGGGTTGGTTTCG |  |  |
| 10 | PT10F | AGGCTTACTACCATACACAT | 43.6 | 1196 |
|  | PT10R | TAGATGGTTAGGTAGAGGAA |  |  |
| 11 | PT11F | CTACTGATCTGCTTCCTACG | 43.6 | 934 |
|  | PT11R | ATCTTTAGCATTGTAGGAGG |  |  |
| 12 | PT12F | CCCTCACACCAATCCTCATA | 43.6 | 1152 |
|  | PT12R | CTGTGTGTGCGTTCGTAGTT |  |  |
| 13 | PT13F | CTGCCTAGCCAACACAAACT | 58 | 1094 |
|  | PT13R | CTCGTCCATATCATCAGCTG |  |  |
| 14 | PT14F | GAAGGAGTGGGCATCATATC | 58 | 1004 |
|  | PT14R | ATTGGTGGAGTTTGTGTTGG |  |  |
| 15 | PT15F | ACACAAACTCCACCAATAAC | 53 | 951 |
|  | PT15R | AGTGTAGGGTGAAGAATCGT |  |  |
| 16 | PT16F | TTCACCCTACACTTCCTCCT | 53 | 1168 |
|  | PT16R | GTTGGTCTTTGGGTCTGTTG |  |  |
| 17 | PT17F | AAAACTACCAACATCCCACC | 53 | 875 |
|  | PT17R | AACCGATTTATGTGCCTGAC |  |  |
| 18 | PT18F | TTTGCGCCTCTGGTTCCTCG | 53 | 1304 |
|  | PT18R | TCGGGCAGGGTTCGGTCTTA |  |  |
