# Supplementary figures and images for: Complete mitogenome of endemic plum-headed parakeet Psittacula cyanocephala – characterization and phylogenetic analysis
Source: PLoS One. 2021 Apr 9;16(4):e0241098. doi: 10.1371/journal.pone.0241098 (PMC8034733; doi:10.1371/journal.pone.0241098)

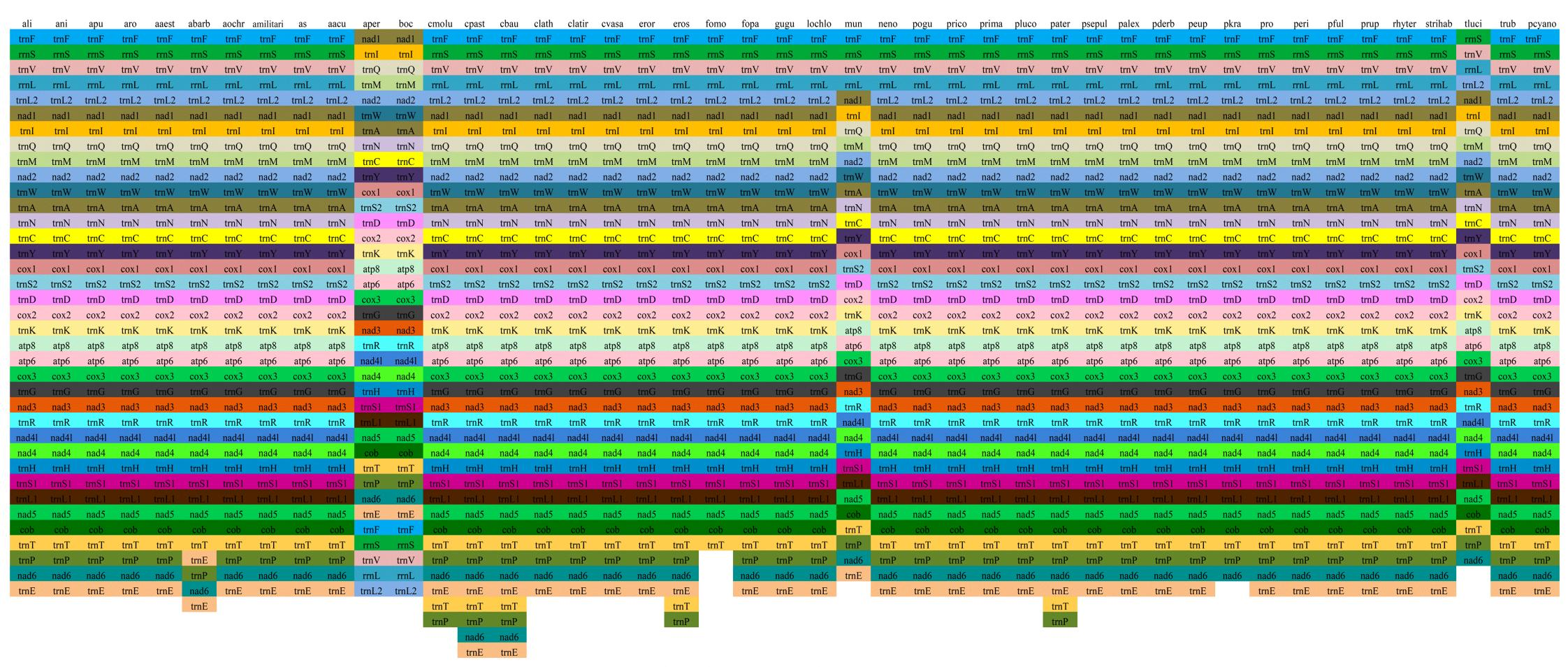

Supplement: S1 Fig — The abbreviations are as follows: ali (Agapornis lilianae), ani (Agapornis nigrigenis),apu (Agapornis pullarius), aro (Agapornis roseicollis), aaest (Amazona aestiva), abarb (Amazona barbadensis), aochr (Amazona ochrocephala), amilitari (Ara militaris), as (Ara severus), aacu (Aratinga acuticaudata), aper (Aratinga pertinax), boc (Brotogeris cyanoptera), cmolu (Cacatua moluccensis), cpast (Cacatua pastinator), cbau (Calyptorhynchus baudinii), clath (Calyptorhynchus lathami), clatir (Calyptorhynchus latirostris), cvasa (Coracopsis vasa), eror (Eclectus roratus), eros (Eolophus roseicapilla), fomo (Forpus modestus), fopa (Forpus passerines), gugu (Guaruba guarouba), lochlo (Lorius chlorocercus), mun (Melopsittacus undulates), neno (Nestor notabilis), pogu (Poicephalus gulielmi), prico (Primolius couloni), prima (Primolius maracana), pluco (Prioniturus lucionensis),pater (Probosciger aterrimus), psepul (Psephotellus pulcherrimus), palex (Psittacula alexandri), pderb (Psittacula derbiana), peup (Psittacula eupatria), pkra (Psittacula krameri), pro (Psittacula roseata), peri (Psittacus erithacus), pful (Psittrichas fulgidus), prup (Pyrrhura rupicola), rhyter (Rhynchopsitta terrisi), strihab (Strigops habroptilus), tluci (Tanygnathus lucionensis), trub (Trichoglossus rubritorquis), pcyano (Psittacula cyanocephala). (DOCX) [file pone.0241098.s007.docx]

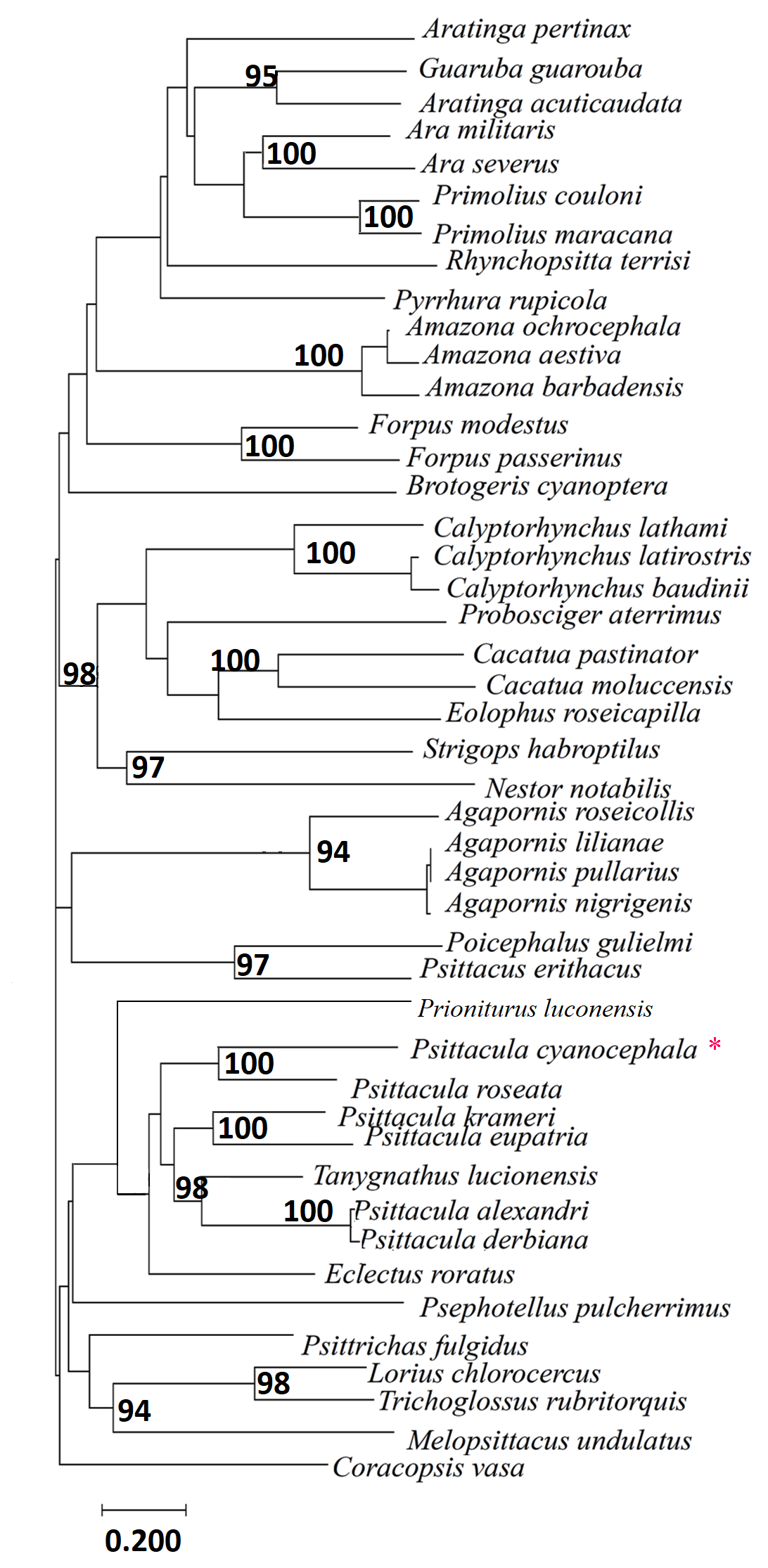

Supplement: S2 Fig — The tree was constructed in Mr.Bayes employing GTR+I+G nucleotide substitution model following 4 independent chains running for 100,000 generations, sub-sampling every 1000 generations and using a burn-in of 100 generations. P. Cyanocephala mitogenome is highlighted with red asterisk mark. (DOCX) [file pone.0241098.s008.docx]
