# Supplementary material for: Complete mitogenome of endemic plum-headed parakeet Psittacula cyanocephala – characterization and phylogenetic analysis
Source: PLoS One. 2021 Apr 9;16(4):e0241098. doi: 10.1371/journal.pone.0241098 (PMC8034733; doi:10.1371/journal.pone.0241098)
Supplement: Attachment — Submitted filename: Response to Reviewers.docx [file pone.0241098.s011.docx]

**Ms. Ref. No.: PONE-D-20-31563R1 - EMID:** **75cc66b576b95812**

**Title: Complete mitogenome of endemic Plum-headed parakeet *Psittacula cyanocephala* – characterization and phylogenetic analysis**

**Response to reviewers**

We would like to thank Dr. Maria Andreína Pacheco (Academic Editor) and the Reviewers for their constructive remarks. We have revised the manuscript as suggested by the Reviewers. The revised manuscript is submitted for further consideration. Please find below a point-by-point rebuttal to the issues raised.

**Additional Editor Comments**:

**Comment 1**: Please, pay attention to the reviewer’s comment and incorporate then in a new version.

**Response:** We have carefully addressed the reviewer’s comment in the new version of the manuscript.

**Reviewer #2**

**Comment 1.** Lines 197-198: “Evolutionary constraints… were…”

**Response**: Sentence rephrased in the manuscript. Please see lines 197-198 on page 10 of file named ‘Manuscript’.

**Comment 2.** Line 205: “The codons used … and were …”

**Response:** Sentence rephrased in the manuscript. Please see lines 205 on page 10 of file named ‘Manuscript’.

**Comment 3.** Lines 197-207: to my knowledge, PAL2NAL is not a software from PAML package, but CODEML is. The authors should revise this.

**Response**: *PAL2NAL* is a programme embedded in PAML. *PAL2NAL* automatically calculates *d*_S_ and *d*_N_ using codeml program in PAML (*http://www.bork.embl.de/pal2nal/#Ref*).

**Comment 4.** Lines 234-235: I insist that the authors should report at least one statistic that they used to check the convergence of the Bayesian chains (average standard deviation of the split frequencies…) and one statistic to check the mixing of the Bayesian chains (ESS…).

**Response**: Thanks for the suggestion. To assess convergence, the average standard deviation of split frequencies was calculated. The effective sample size value of the trace was also diagnosed to confirm the mixing of the Bayesian Markov chains. Please see lines 236-239 on page 11, 12 of file named ‘Manuscript’. The resulting values and data are submitted as additional supplementary information.

**Comment 5.** Line 415: “The evolutionary rate…”

**Response:** Sentence rephrased in the manuscript. Please see line 410 on page 22 of file named ‘Manuscript’.

**Comment 6.** The alignment data as well as the phylogenetic trees (nwk/nexus) should be made available (at the supporting material or a public repository).

**Response**: The alignment file, both BI and ML tree files are submitted as additional supplementary information.

**Reviewer #3**

**Comment 1.** The authors did a really good job improving the manuscript by addressing all the previous reviewers’ concerns. The manuscript is mainly focused on the building and characterization of the genome of Psittacula cyanocephala, and this is the main strength of it. Phylogenetic analyses, however, as mentioned by one of the previous reviewers, did not provide any new insights on Psittacula parakeets phylogenetic relationships, even thought show support to previous claims on Psittacula paraphyly. The sampling scheme of the includes a limited number of taxa because of the few available mitogenomes (6 of the 16 Psittacula species and only 1 of the 4 Tanygnathus species) and because of this, it only shows the potential applications of the use of mitogenomes for phylogenetic analyses. Phylogenetic analyses also include data from 44 parrot species but these do not provide new information on the relationships within Psittaciformes.

**Response**: Thank you very much for your thoughtful comments and insight. The complete mitogenome of *Psittacula cyanocephala* is necessary to build the complete phylogeny of *Psittacula* genus complex. Our work does lend support to paraphyly within *Psittacula* genus as indicated by previous studies. Evidences from multiple sources (complete mitogenomes, single nuclear/mitochondrial genes etc.) are necessary to propose and validate a comprehensive phylogeny (Tietze DT, 2018). We believe our study is an important contribution in this aspect.

- Tietze DT. Bird species: how they arise, modify and vanish. Springer Nature; 2018.
